# Associations of serum sTREM-1 and sTREM-2 with mortality and neurological prognosis in patients resuscitated from cardiac arrest: a machine learning-based approach

**DOI:** 10.3389/fmed.2026.1717571

**Published:** 2026-03-03

**Authors:** Ling Wang, Peiyan Chen, Yushu Chen, Zheyuan Fan, Dongping Yu, Wei Zhang, Bao Fu, Ping Gong

**Affiliations:** 1 Department of Critical Care Medicine, Affiliated Hospital of Zunyi Medical University, Zunyi, Guizhou, China; 2 Department of Emergency Medicine, First Affiliated Hospital of Dalian Medical University, Dalian, Liaoning, China; 3 Department of Thoracic Surgery, Affiliated Hospital of Zunyi Medical University, Zunyi, Guizhou, China; 4 Department of Emergency Medicine, Second Affiliated Hospital of Dalian Medical University, Dalian, Liaoning, China; 5 Department of Emergency Medicine, Shenzhen People’s Hospital (The Second Clinical Medical College, Jinan University; The First Affiliated Hospital, Southern University of Science and Technology), Shenzhen, Guangdong, China

**Keywords:** cardiac arrest, machine learning, prognosis, sTREM-1, sTREM-2

## Abstract

**Background:**

Patients resuscitated from cardiac arrest (CA) commonly have poor outcomes with a high mortality rate. We aimed to determine the predictive values of serum soluble triggering receptor expressed on myeloid cells 1 and 2 (sTREM-1 and sTREM-2) in patients after return of spontaneous circulation (ROSC) and to develop machine learning (ML) prediction models.

**Methods:**

We prospectively enrolled adult CA patients successfully resuscitated after cardiopulmonary resuscitation between November, 2021 to December, 2023. Serum sTREM-1, sTREM-2 and other biomarkers were measured on days 1, 3 and 5 after ROSC. The primary outcome was 28-day all-cause mortality. The secondary outcome was 3-month neurological prognosis. The performance of serum sTREM-1, sTREM-2, as well as the developed ML prediction models, to predict 28-day all-cause mortality and 3-month neurological prognosis were studied.

**Results:**

The study enrolled 120 patients, including 32 survivors and 88 non-survivors, with 30 healthy volunteers. Both sTREM-1 and sTREM-2 levels increased in patients after ROSC, with a larger increase in the non-survivors than survivors. Moreover, eleven features, including sTREM-1 and sTREM-2, were ultimately identified to build ML models. Among other ML models, the eXtreme Gradient Boosting (XGBoost) and Random Forest (RF) models showed strong performances for predicting 28-day all-cause mortality and 3-month neurological prognosis, respectively.

**Conclusion:**

Serum sTREM-1 performed better than sTREM-2 to predict mortality and neurological outcome after ROSC. Furthermore, the newly developed XGBoost and RF models incorporating sTREM-1 and/or sTREM-2 demonstrated superior predictive accuracy compared to conventional clinical scoring systems.

## 1 Introduction

Patients with cardiac arrest (CA) commonly have poor outcomes. The post-CA survival rates to hospital discharge were 10–30% in Europe and the United States (1), but only approximately 1.15% in China (2). Following return of spontaneous circulation (ROSC), patients undergo complex pathophysiological changes known as the post-cardiac arrest syndrome (PACS) (3). The PACS is characterized by an early inflammatory response, accompanied by circulatory dysfunction and hypoxic brain injury (3). Notably, the inflammatory response triggered by ischemic/reperfusion (I/R) could contribute to the secondary brain injury and high mortality (4, 5). Therefore, activated circulating cytokines and inflammatory mediators after ROSC may serve as promising prognostic biomarkers.

Triggering receptors expressed on myeloid cells (TREM)-1 and 2, members of the immunoglobulin superfamily, are predominantly localized on the membrane of myeloid-origin cells, including monocytes/macrophages, neutrophils and microglia (6). TREM-1 and TREM-2 are cell surface receptors involved in various cellular processes, such as neurological development and inflammatory responses (6–8). Mechanistically, TREM-1 can trigger the expressions of proinflammatory cytokines interleukin-6 (IL-6), interleukin-1β, and tumor necrosis factor-*α* (TNF-α) after binding to the adaptor DNAX activation protein-12 (DAP-12). In addition, TREM-1 synergistically amplifies toll-like receptor (TLR) 4 or TLR2 driven inflammatory responses (9, 10). In the brain, TREM-1 is primarily localized on the surface of microglia and functions as a potent neuroinflammation amplifier (11). Unlike TREM-1, TREM-2 associates with DAP12 and cross-talks with TLR signaling pathways to ameliorate the pro-inflammatory cytokine secretion (7, 12–15).

The origin of soluble TREM molecules (sTREM) is likely derived after the proteolysis of the membrane-bound TREM by matrix metalloproteinases (MMP), such as matrix metalloproteinase-9 (MMP-9) (16, 17). Simultaneously with cell surface induction and upregulation of TREM-1 and TREM-2 expressions, sTREM-1 and sTREM-2 may accumulate in the circulation during inflammation and tissue injury (17–19). Recently, several studies have demonstrated the relevant prognostic value of sTREM-1 in both infectious or non-infectious diseases, including sepsis, myocardial infarction, and cardiogenic shock (8, 19, 20). Similarly, cerebrospinal fluid or serum sTREM-2 may provide a valuable biomarker for neuroinflammatory and neurodegenerative diseases (16, 21, 22). However, serum levels of sTREM-1 and sTREM-2 in CA patients after ROSC and their association with 28-day all-cause mortality and 3-month neurological prognosis remain unclear.

In recent years, machine learning (ML) technology has exhibited superior data processing capabilities with improved precision in predictive disease outcomes. Moreover, contemporary ML models are gaining recognition as robust alternatives to conventional statistical approaches such as logistic regression or cox proportional hazards models for outcome prediction (23, 24). While ML algorithms have been applied to predict outcomes after CA, the specific integration of the novel inflammatory biomarkers sTREM-1 and sTREM-2 with ML models remains underexplored (25, 26). Here, we conducted a prospective cohort study employing ML algorithms to evaluate the prognostic predictive role of serum sTREM-1 and sTREM-2 in post-CA patients.

## 2 Methods

### 2.1 Study design and participant selection

This prospective cohort study consecutively enrolled adult CA patients admitted to the cardiac intensive care unit (CICU) and emergency intensive care unit (EICU) at the First Affiliated Hospital of Dalian Medical University (Dalian, China) between November 2021 and December 2023. The study protocol was approved by the Medical Ethics Committee at the First Affiliated Hospital of Dalian Medical University (PJ-KS-KY-2022-251) and registered as MR-21-23-034771 in the Medical Research Registration. Written informed consent was obtained from all patients or their healthcare proxies. All study procedures were in compliance with the guidelines of the Declaration of Helsinki.

The inclusion criteria were CA patients with, (1) age ≥18 years old and (2) successful ROSC after cardiopulmonary resuscitation (CPR). The exclusion criteria were patients with, (1) sepsis, autoimmune disorders, past history of cancer, recent or long-term use of corticosteroids, (2) hematological diseases, malignancies, neurological deficits, (3) pregnancy, lactation, or (4) incomplete information. We also enrolled sex- and age-matched healthy volunteers as a control group. All patients received standard post-ROSC management throughout the study period, including multimodal neuro-prognostication, according to the 2020 International Consensus on CPR (27).

### 2.2 Data collection

We collected the clinical data, including demographics, causes of CA, medical history, bystander CPR, initial heart rhythm, CPR time, length of ICU stays, laboratory results, and outcomes, from the electronic medical record system. The Acute Physiology and Chronic Health Evaluation (APACHE II) score and Sequential Organ Failure Assessment (SOFA) score were calculated upon ICU admission. The primary outcome was 28-day all-cause mortality. The secondary outcome was 3-month neurological prognosis that was assessed by a healthcare professional who was blinded to all biomarker results, using cerebral performance category (CPC) score (1–2 and 3–5 referred as favorable and poor outcomes, respectively).

To ensure clarity, we explicitly defined “CPR time” as the total resuscitation duration, measured from the initiation of chest compressions to sustained ROSC. The documentation of initial rhythm was obtained from either emergency medical services records or upon the patient’s arrival at the receiving emergency department.

### 2.3 Measurement of biomarkers

The peripheral venous blood samples were drawn from patients at 24 h, 72 h and 120 h after ROSC or healthy volunteers after enrollment. Only samples collected within 6 hours of the scheduled times were considered for statistical analysis. Enzyme-linked immunosorbent assay (ELISA) kits were used to determine the levels of sTREM-1 (Elabscience, Wuhan, China); sTREM-2, sTLR-4, and MMP-9 (MyBioSource, San Diego, CA, United States); NSE (CUSABIO, Wuhan, China); and IL-6, IL-10, TNF-*α*, and HMGB-1 (Elabscience, Wuhan, China), following the manufacturers’ instructions.

Laboratory personnel were blinded to the participant clinical information.

### 2.4 Model development, evaluation and interpretation

We conducted least absolute shrinkage and selection operator (LASSO) regression analyses to optimize the screening variables related to prognosis in CA patients. This approach enhances the prediction precision of the ML models and safeguards against overfitting. All input variables—including demographic data, clinical parameters, laboratory results, and biomarker levels (sTREM-1 and sTREM-2)—were restricted to those measured or reliably ascertainable within the first 24 h following ROSC.

The cohort data were partitioned through stratified random sampling into training (70%) and test (30%) subsets for model development and predictive validation, respectively. A total of eight ML algorithms were included for model construction for predicting 28-day all-cause mortality and 3-month neurological prognosis. These algorithms were Logistic Regression (LR), Random Forest (RF), Gaussian Naive Bayes (GNB), Support Vector Machine (SVM), K-Nearest Neighbor (KNN), eXtreme Gradient Boosting (XGBoost), Light Gradient Boosting Machine (LightGBM), and Decision Tree (DT). We incorporated key methodological principles from the recent guideline on ICU big-data and ML research (28). A rigorous methodology integrating internal fivefold cross-validation and grid search was implemented to discern the most suitable hyperparameters for each distinct model, significantly improving the prediction accuracy in each modeling architecture. Optimal Hyperparameter Configurations for Machine Learning Models, which details the final tuned hyperparameters for each algorithm evaluated was included in Supplementary Table 1. The Shapley Additive explanation (SHAP) method was implemented to interpret predictions generated by ML models, helping to quantify feature importance and deconstruct individual feature contributions through interactive visualizations. The performance of models was comprehensively evaluated by the area under the receiver operating characteristic curve (AUROC), precision-recall (PR) curve, accuracy, sensitivity, specificity, positive predictive value (PPV), negative predictive value (NPV), F1 score, Kappa value, and clinical utility quantification via calibration curve and decision curve analysis (DCA).

### 2.5 Statistical analysis

Statistical analyses were performed in GraphPad Prism (version 9, GraphPad Software Inc., CA, United States), and Python (version 3.12). Based on preliminary sTREM-1 data (17.85 ± 3.77 *vs* 167.90 ± 36.79 *vs* 327.90 ± 58.47 pg./mL in healthy control, survivors, and non-survivors, respectively) and sTREM-2 data (321.19 ± 37.11 *vs* 133.60 ± 279.02 *vs* 33.31 ± 368.27 pg./mL in healthy control, survivors, and non-survivors, respectively), a sample size of 25 patients (*α* = 0.05, power = 80%, dropout rate of 20%) per group was required. The continuous variables were all nonnormally distributed and described as median (interquartile range), while categorical variables were described as counts (percentage). For multiple comparisons, non-parametric analyses were conducted by Friedman and Kruskal–Wallis tests with a Dunn’s test for the *post hoc* pairwise analysis, or Mann–Whitney U test for two-group comparisons. The categorical variables were compared by Pearson Chi-squared test or Fisher exact test. Bivariate correlation analyses were conducted using Spearman’s correlation coefficient. The prediction models were built implementing eight ML algorithms. A two-sided *p* < 0.05 was considered statistically significant difference.

## 3 Results

### 3.1 Participant enrollment and baseline characteristics

**Figure 1:**
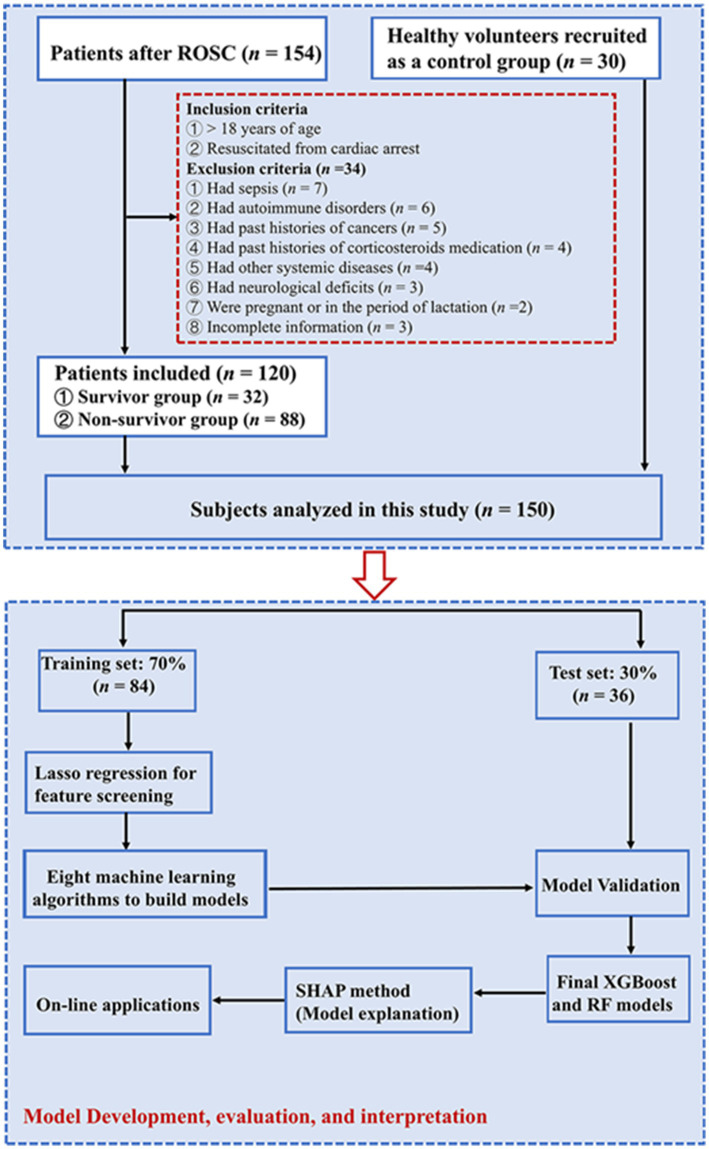
Participant enrollment flowchart. RF, Random Forest; ROSC, restoration of spontaneous circulation; SHAP, Shapley Additive explanation; XGBoost, eXtreme Gradient Boosting.

A total of 154 CA patients and 30 healthy controls were screened, with 120 CA patients meeting the eligibility criteria (see Figure 1). There were 32 (26.7%) and 88 (73.3%) patients in the survival and non-survival groups, respectively. Baseline characteristics across groups are summarized in Table 1. Age, sex, medical history, etiology of arrest, the location of arrest, and main treatments showed no significant intergroup differences (all *p* > 0.05). However, non-survivors exhibited lower rates of bystander CPR (50.0% *vs* 87.5%) and shockable initial rhythms (33.0% *vs* 56.3%) compared to survivors (both *p* < 0.05). Moreover, compared with the survivors, the non-survivors experienced longer CPR duration, shorter length of ICU-stay and higher severity scores (SOFA and APACHE II scores) compared with survivors (both *p* < 0.05). CA patients had higher levels of white blood cell, neutrophils, procalcitonin, C-reactive protein (CRP), lactate, aspartate aminotransferase (AST), alanine aminotransferase (ALT), creatinine, brain natriuretic peptide, and high-sensitivity troponin I than healthy volunteers (all *p* < 0.05). In addition, serum AST, ALT and lactate were significantly increased in non-survivors than survivors (all *p* < 0.05).

**Table 1:** Comparisons of baseline characteristics between survivors and non-survivors.

Characteristics	Healthy volunteers	Survivors	Non-survivors	*P*
	(*n* = 30)	(*n* = 32)	(*n* = 88)	
Age, years	62.5 (49.5, 75.5)	69.0 (57.0, 79.0)	69.0 (58.3, 79.0)	0.291
Male, *n* (%)	19 (63.3%)	20 (62.5%)	53 (60.2%)	0.944
Past medical history, *n* (%)				
Diabetes	–	9 (28.1%)	21 (23.9%)	0.634
Coronary heart disease	–	5 (15.6%)	16 (18.2%)	0.744
Hypertension	–	17 (53.1%)	48 (54.5%)	0.890
Chronic kidney disease	–	3 (9.3%)	8 (9.1%)	0.962
Chronic pulmonary disease	–	6 (18.8%)	15 (17.0%)	0.828
Post-operation	–	5 (15.6%)	14 (15.9%)	0.970
Cardiac arrest cause, *n* (%)				0.834
Respiratory	–	10 (31.3%)	21 (23.9%)	–
Cardiac	–	10 (31.3%)	34 (38.6%)	–
Cerebral	–	5 (15.6%)	13 (14.8%)	–
Others	–	7 (21.8%)	20 (22.7%)	–
Cardiac arrest location, *n* (%)				0.242
Out of hospital	–	8 (25.0%)	32 (36.4%)	–
In hospital	–	24 (75.0%)	56 (63.6%)	–
Bystander CPR, *n* (%)	–	28 (87.5%)	44 (50.0%)^a^	<0.001
Initial cardiac rhythm, *n* (%)				0.009
Shockable rhythm	–	18 (56.3%)	29 (33.0%)^a^	–
Non-shockable rhythm	–	14 (43.7%)	59 (67.0%)^a^	–
CPR time, min	–	8.0 (5.0, 11.8)	16.0 (8.0, 28.8)^a^	<0.001
Length of ICU stay, days	–	14.5 (11.3, 18.0)	4.0 (1.0, 8.0)^a^	<0.001
Cause of death, *n* (%)				
Refractory hemodynamic shock	–	–	24 (27.3%)	–
Neurological withdrawal of care	–	–	16 (18.3%)	–
Comorbid withdrawal of care	–	–	13 (14.7%)	–
Multiple organ dysfunction	–	–	14 (15.9%)	–
Sudden cardiac death	–	–	13 (14.7%)	–
Respiratory failure	–	–	8 (9.1%)	–
Treatments, *n* (%)				
Renal replacement therapy	–	9 (28.1%)	30 (34.1%)	0.381
Mechanical ventilation	–	30 (93.8%)	88 (100%)	0.069
Hemodynamic support	–	30 (93.8%)	88 (100%)	0.069
Laboratory findings				
White blood cell, ×10^9^/L	6.83 (5.46, 7.92)	12.79 (9.20, 20.91)^b^	14.40 (9.79, 19.56)^b^	<0.001
Neutrophils, ×10^9^/L	4.36 (3.28, 5.12)	9.39 (6.49, 16.78)^b^	10.41 (6.57, 15.56)^b^	<0.001
C-reactive protein, mg/dL	0.23 (0.09, 0.32)	1.66 (0.69, 2.48)^b^	2.04 (1.20, 3.67)^b^	<0.001
Procalcitonin, ng/mL	0.21 (0.08, 0.31)	1.39 (0.08, 5.72)^b^	0.68 (0.12, 8.94)^b^	0.001
Creatinine, μmol/L	72.00 (60.75, 80.25)	88.00 (66.75, 116.00)^b^	104.50 (78.50, 171.75)^b^	<0.001
AST, IU/L	32.00 (20.50, 39.25)	53.00 (29.00, 157.00)^b^	108.00 (47.25, 228.25)^a^^b^	<0.001
ALT, IU/L	24.50 (17.75, 34.25)	42.50 (16.25, 116.50)^b^	67.50 (40.00, 149.25)^a^^b^	<0.001
Lactate, mmol/L	0.23 (0.14, 0.52)	3.27 (1.54, 5.73)^b^	5.79 (3.31, 9.54)^a^^b^	<0.001
Hs-TnI, μg/L	0.001 (0.000, 0.010)	1.390 (0.483, 4.618)^b^	2.930 (0.533, 9.793)^b^	<0.001
BNP, pg./mL	28.54 (20.68, 44.61)	176.62 (67.22, 797.62)^b^	317.35 (82.83, 965.26)^b^	<0.001
APACHE II score	–	19.5 (11.0, 24.0)	22.0 (17.0, 26.8)^a^	0.029
SOFA score	–	5.0 (2.0, 8.7)	8.0 (5.0, 9.0)^a^	0.019
3-month CPC (1–2), *n* (%)		21 (65.6%)		

Data are presented as median (interquartile range) or *n* (percentile), unless specified otherwise.

a *P* < 0.05 compared with survivors.

b *P* < 0.05 compared with healthy volunteers.

APACHE II, Acute Physiology and Chronic Health Evaluation II; ALT, alanine aminotransferase; AST, aspartate aminotransferase; BNP, brain natriuretic peptide; CPC, cerebral performance category; CPR, cardiopulmonary resuscitation; Hs-TnI, High sensitivity troponin I; ICU, intensive care unit; SOFA, Sequential Organ Failure Assessment.

Comparisons between CA patients with favorable (CPC 1–2) and poor (CPC 3–5) neurological outcomes were detailed in Supplementary Table 2. Patients with poor outcomes received mechanical ventilation and hemodynamic support therapy significantly more frequently than those with favorable outcomes (all *p* < 0.05). Poor outcomes were associated with reduced bystander CPR rates and shockable rhythms, prolonged CPR duration and length of ICU-stay, higher severity scores (SOFA and APACHE II scores), and elevated AST, ALT and lactate levels (all *p* < 0.05).

### 3.2 Comparisons of serum sTREM-1, sTREM-2, sTLR-4, MMP-9, NSE, and inflammatory cytokines

**Figure 2:**
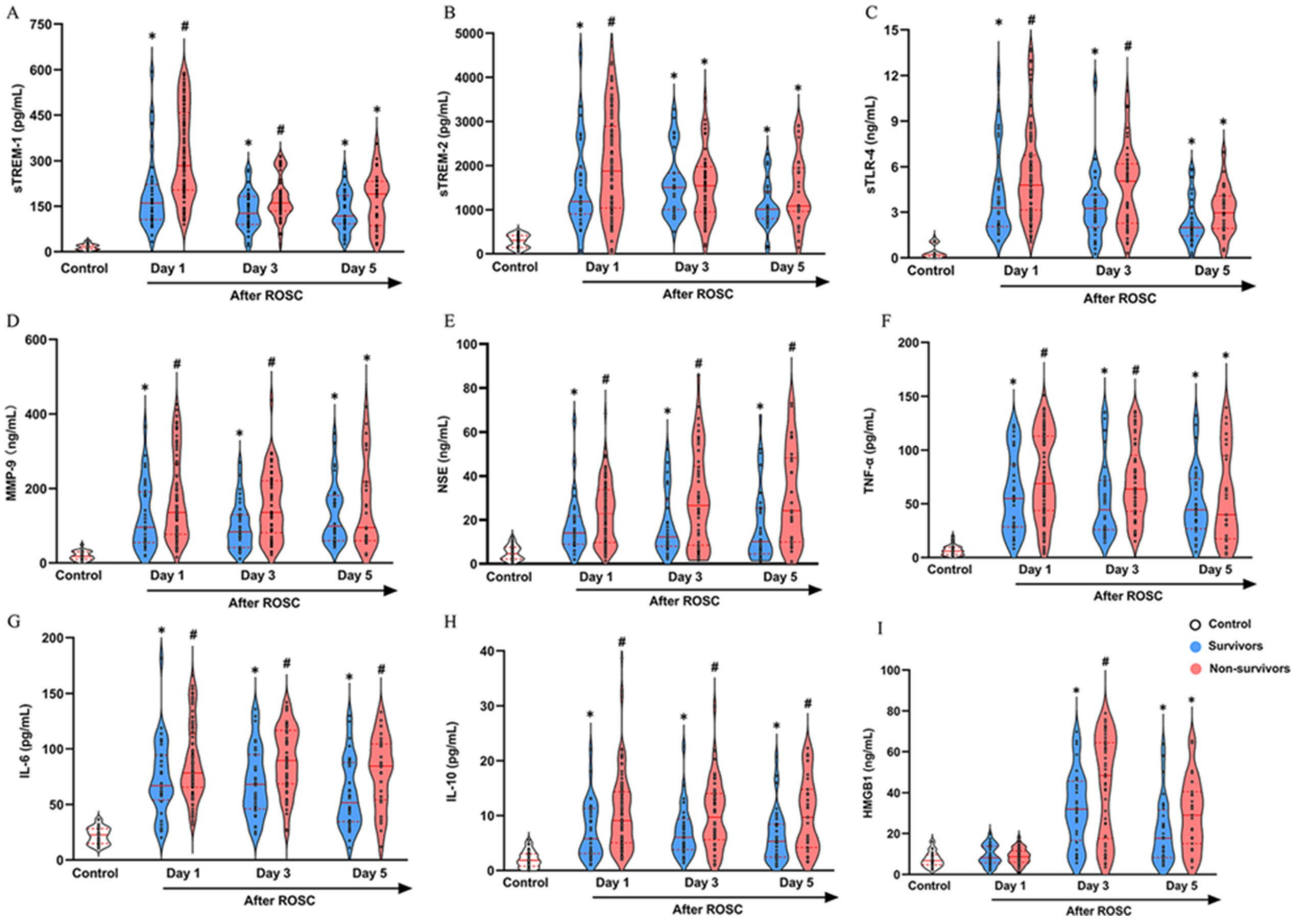
Serum levels of sTREM-1 **(A)**, sTREM-2 **(B)**, sTLR-4 **(C)**, MMP-9 **(D)**, NSE **(E)**, TNF-*α* **(F)**, IL-6 **(G)**, IL-10 **(H)**, and HMGB1 **(I)** in cardiac arrest patients. ^*^ *p* < 0.05 compared with healthy volunteers; ^#^ *p* < 0.05 compared with survivors. HMGB1, high mobility group protein 1; IL-6, Interleukin-6; IL-10, Interleukin-10; MMP-9, matrix metalloproteinase-9; NSE, neuron-specific enolase; ROSC, restoration of spontaneous circulation; sTREM-1, soluble triggering receptors expressed on myeloid cells 1; sTREM-2, soluble triggering receptors expressed on myeloid cells 2; sTLR-4, soluble toll-like receptor 4; TNF-α, tumor necrosis factor-α.

Patients had significantly higher post-ROSC serum levels of sTREM-1, sTREM-2, sTLR-4, MMP-9, NSE, IL-6, TNF-*α*, and IL-10 on post-ROSC day 1, 3, and 5 as well as serum HMGB1 on post-ROSC day 3 and 5 compared with healthy volunteers (all *p* < 0.05, Figure 2). Serum sTREM-1, sTLR-4, MMP-9 and TNF-α on post-ROSC day 1 and 3, serum sTREM-2 on post-ROSC day 1, serum HMGB1 on post-ROSC day 3, and serum NSE, IL-6 and IL-10 on post-ROSC day 1, 3, and 5 were significantly increased in non-survivors than those in survivors (all *p* < 0.05).

Comparisons of biomarkers between CA patients with favorable and poor neurological outcomes were showed in Supplementary Table 3. Serum sTREM-1, sTREM-2, sTLR-4 on post-ROSC day 1, and serum MMP-9 and NSE on post-ROSC day 1 and 3 were significantly increased in patients with poor outcomes than those with favorable outcomes (all *p* < 0.05).

### 3.3 Correlations of serum sTREM-1 and sTREM-2 with other serum cytokines, SOFA score, and APACHE II score

**Figure 3:**
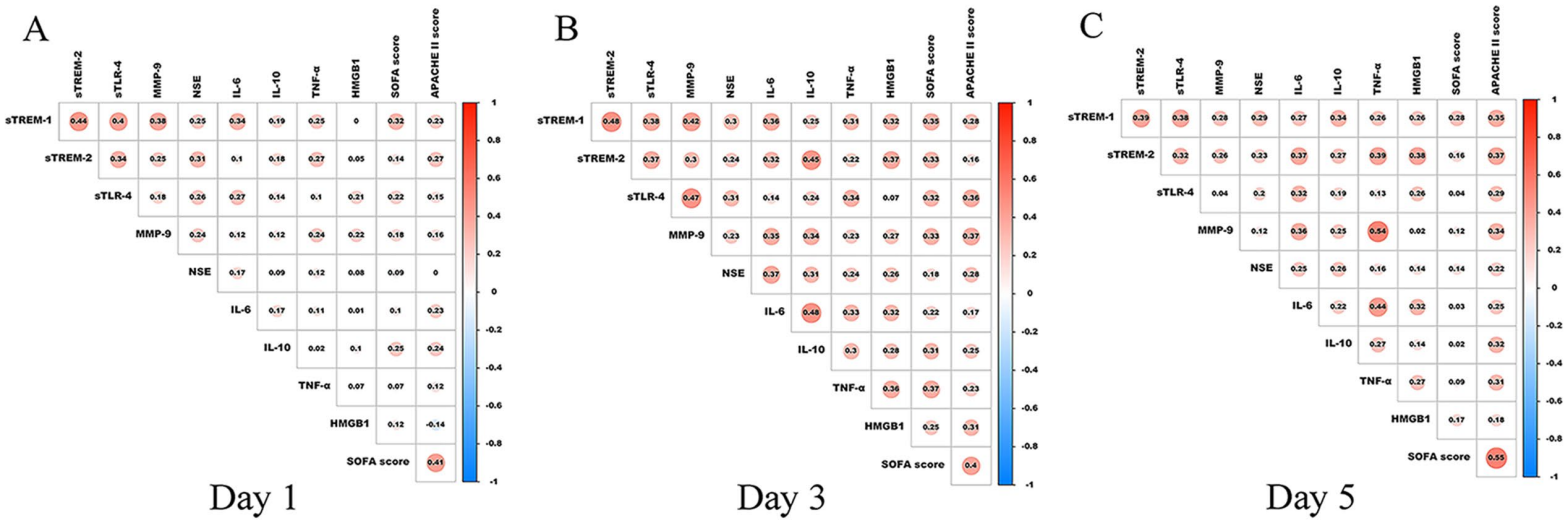
Heatmaps of correlation of serum sTREM-1 and sTREM-2 with other variables in cardiac arrest patients. APACHE II, Acute Physiology and Chronic Health Evaluation; HMGB1, high mobility group protein 1; IL-6, Interleukin-6; IL-10, Interleukin-10; MMP-9, matrix metalloproteinase-9; NSE, neuron-specific enolase; sTREM-1, soluble triggering receptors expressed on myeloid cells 1; sTREM-2, soluble triggering receptors expressed on myeloid cells 2; sTLR-4, soluble toll-like receptor 4; SOFA, Sequential Organ Failure Assessment.

Serum sTREM-1 and sTREM-2 were positively correlated with sTLR-4, MMP-9, NSE, IL-6, IL-10, TNF-α, APACHE II, and SOFA on post-ROSC day 1, 3, and 5 and with HMGB1 on post-ROSC day 3 and 5 (see Figure 3).

### 3.4 Feature selection

**Figure 4:**
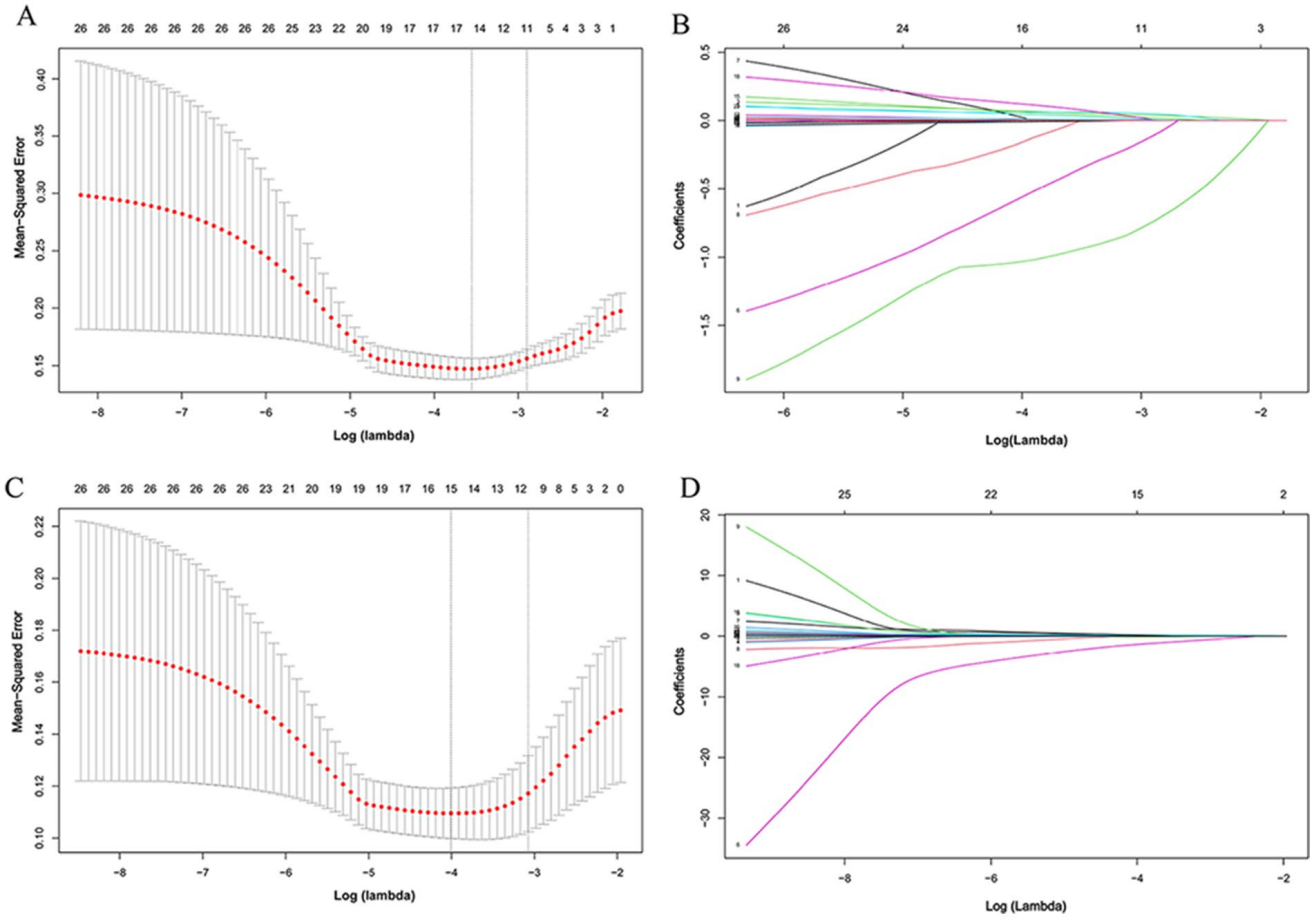
Predictor selection of 28-day all-cause mortality **(A,B)** and 3-month neurological prognosis **(C,D)** based on LASSO-logistic regression analysis. LASSO, least absolute shrinkage and selection operator.

Considering multi-collinearity among the included variables, LASSO regression analysis was implemented for feature selection. The analysis evaluated 26 candidate variables (see Supplementary Table 4). The clinical data collected in the first 24 h after ICU admission were served as model inputs. LASSO regression identified eleven predictive variables (lambda.lse = 0.0551) with nonzero coefficients for 28-day all-cause mortality (see Figures 4A,B). These eleven features were CPR time, SOFA score, Initial cardiac rhythm, Bystander CPR, sTREM-1, NSE, IL-6, IL-10, CRP, hs-TnI and Creatinine. With a lambda.lse of 0.0461, eleven features were included (CPR time, SOFA score, Initial cardiac rhythm, Bystander CPR, sTREM-1, sTREM-2, NSE, IL-6, lactate, WBC and hs-TnI) for predicting 3-month neurological prognosis (see Figures 4C,D).

### 3.5 Development, evaluation, and comparison of the predictive models

**Figure 5:**
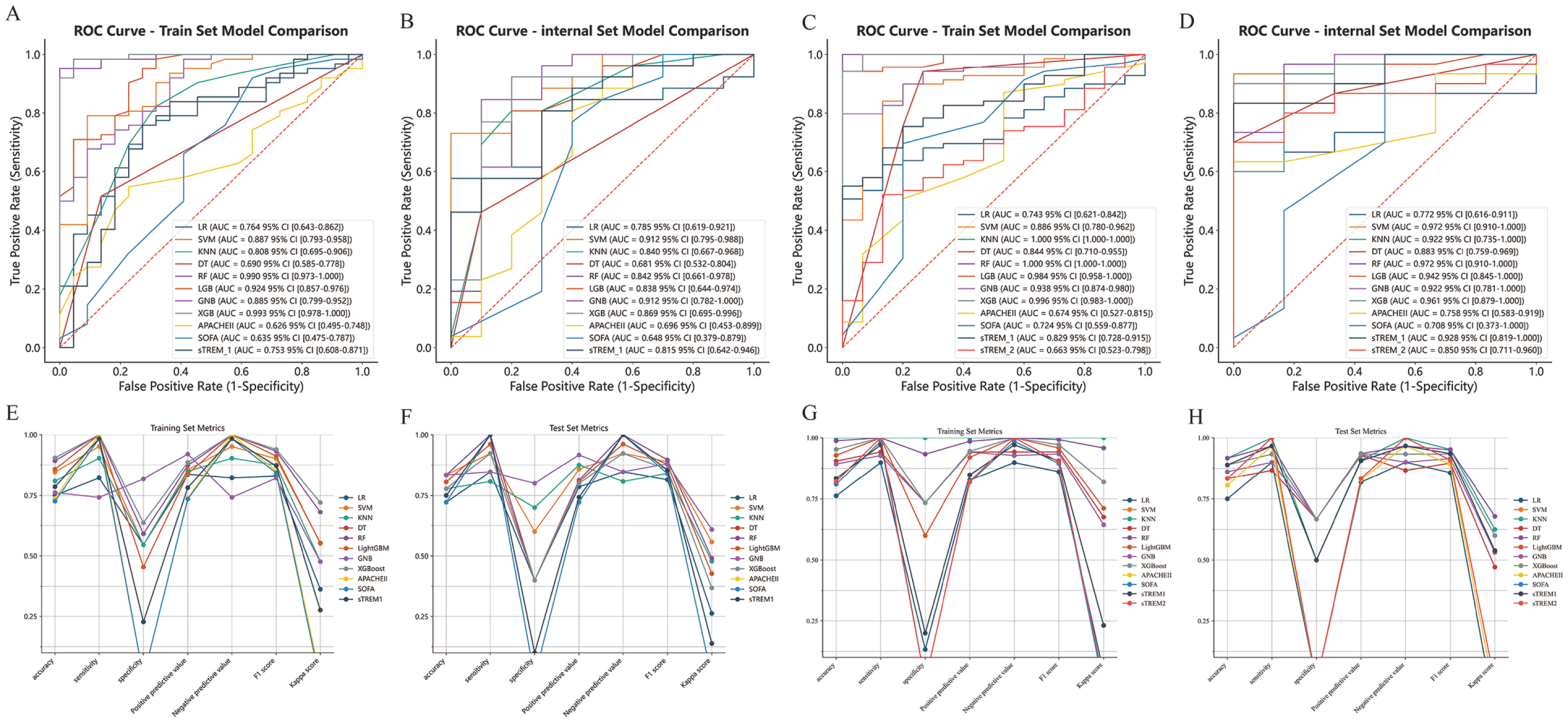
Predictive performance and comparison of eight models, SOFA score, APACHE II Score, sTREM-1, and sTREM-2 for 28-day all-cause mortality **(A,B,E,F)** and 3-month neurological prognosis **(C,D,G,H)**. **(A,C)** The training set ROC curve; **(B,D)** The test set ROC curve; **(E,G)** Evaluation metrics for the training set; **(F,H)** Evaluation metrics for the test set. AUC, area under the curve; APACHE II, Acute Physiology and Chronic Health Evaluation; DT, Decision Tree; GNB, Gaussian Naive Bayes; KNN, K-Nearest Neighbor; LGB/LightGBM, Light Gradient Boosting Machine; LR, Logistic Regression; RF, Random Forest; ROC, receiver operating characteristic; SOFA, Sequential Organ Failure Assessment; SVM, Support Vector Machine; sTREM-1, soluble triggering receptors expressed on myeloid cells 1; sTREM-2, soluble triggering receptors expressed on myeloid cells 2; XGB/XGBoost, eXtreme Gradient Boosting.

Model inputs comprised the afore-mentioned LASSO-selected features. The baseline characteristics of the training and test sets were listed in Tables 2, 3. To predict 28-day all-cause mortality and 3-month neurological prognosis, ROC curves were generated from the eight ML models (see Figure 5). Comprehensive performance metrics detailing the predictive capabilities of the ML models across the prognostic outcomes were delineated in the Supplementary Tables 5, 6. Taking each metric of the models into account, XGBoost and RF were found to be the most proficient in predicting 28-day all-cause mortality and 3-month neurological prognosis, respectively. When predicting 28-day all-cause mortality in CA patients, XGBoost model has a high AUROC of 0.993 (95% confidence interval, CI, 0.976–1.000), with accuracy 90.5%, sensitivity 100%, specificity 63.6%, PPV 88.6%, NPV 100%, F1 score 93.9%, and Kappa score 72.1% in the training set. Meanwhile, the performance of the XGBoost model remained stable in the internal validation set (AUC: 0.873; 95% CI 0.679–1.000). When predicting 3-month neurological prognosis, RF model consistently outperformed its counterparts across all evaluated metrics. As described in Supplementary Table 6, the training set AUROC value, accuracy, sensitivity, specificity, PPV, NPV, F1 score, and Kappa score were 1.000 (95% CI 1.000–1.000), 98.8, 100, 93.3, 98.6, 100, 99.3, and 95.8%, respectively, with the AUROC of 0.994 (95%CI 0.970–1.000), in the internal validation set. The PR curves, calibration curves and DCA curves showed that the XGBoost and RF models provided significantly higher net gains than the baseline strategy in predicting two outcomes (Supplementary Figure S1). The confusion matrix showed the difference in the model’s performance on different datasets (Supplementary Figure S1). We also compared the performances of the serum TREM-1, sTREM-2, and other variables in ML models (Figure 5).

**Table 2:** Comparisons of characteristics of study in the training and test cohorts to predict 28-day all-cause mortality.

Characteristics	Overall	Training set	Test set	*P*
	(*n* = 120)	(*n* = 84)	(*n* = 36)	
CPR time, min	14.0 (7.0, 25.0)	14.0 (7.0, 28.0)	11.5 (8.0, 24.0)	0.788
Bystander CPR, *n* (%)	72 (60%)	49 (58.3%)	23 (63.9%)	0.685
Initial cardiac rhythm, *n* (%)				0.839
Shockable rhythm	47 (39.2%)	32 (38.1%)	15 (41.7%)	–
Non-shockable rhythm	73 (60.8%)	52 (61.9%)	21 (58.3%)	–
SOFA score	8.0 (4.3, 9.0)	7.0 (4.0, 9.0)	8.0 (5.3, 9.0)	0.124
sTREM-1, pg./mL	229.78 (164.41, 417.91)	234.40 (161.82, 421.54)	223.01 (175.40, 352.16)	0.819
NSE, ng/mL	19.23 (9.34, 30.94)	21.29 (9.68, 31.22)	16.68 (9.06, 29.24)	0.277
IL-6, pg./mL	75.98 (60.12, 104.92)	79.40 (61.28, 107.82)	67.77 (57.74, 94.31)	0.111
IL-10, pg./mL	8.72 (4.32, 13.18)	8.93 (4.31, 13.67)	8.52 (4.37, 12.94)	0.352
C-reactive protein, mg/dL	1.89 (1.15, 2.96)	1.93 (1.22, 2.95)	1.83 (1.04, 2.98)	0.516
Creatinine, μmol/L	98.00 (76.50, 157.50)	96.00 (76.50, 154.75)	102.50 (76.00, 161.75)	0.781
Hs-TnI, μg/L	1.66 (0.53, 8.81)	2.00 (0.38, 9.08)	1.47 (0.58, 7.73)	0.761
Non-survivors, *n* (%)	88 (73.3%)	62 (73.8%)	26 (72.2%)	0.857

Data are presented as median (interquartile range) or *n* (percentile), unless specified otherwise. CPR, cardiopulmonary resuscitation; Hs-TnI, High sensitivity troponin I; IL-6, interleukin-6; IL-10, interleukin-10; NSE, neuron-specific enolase; SOFA, Sequential Organ Failure Assessment; sTREM-1, soluble triggering receptors expressed on myeloid cell-1.

**Table 3:** Comparisons of characteristics of study in the training and test cohorts to predict 3-month neurological prognosis.

Characteristics	Overall	Training set	Test set	*P*
	(*n* = 120)	(*n* = 84)	(*n* = 36)	
CPR time, min	14.0 (7.0, 25.0)	15.0 (8.0, 28.0)	9.5 (5.3, 16.0)	0.017
Bystander CPR, *n* (%)	72 (60.0%)	51 (60.7%)	21 (58.3%)	0.841
Initial cardiac rhythm, *n* (%)				
Shockable rhythm	47 (39.2%)	32 (38.1%)	15 (41.7%)	0.655
Non-shockable rhythm	73 (60.8%)	52 (61.9%)	21 (58.3%)	0.499
SOFA score	8.0 (4.3, 9.0)	7.0 (4.0, 9.0)	8.0 (5.0, 9.0)	0.411
sTREM-1, pg./mL	229.78 (164.41, 417.91)	234.40 (169.78, 446.63)	221.17 (135.67, 350.25)	0.327
sTREM-2, pg./mL	1678.3 (1008.2, 2694.6)	1596.0 (972.2, 2694.6)	1696.9 (1058.4, 3027.8)	0.602
NSE, ng/mL	19.23 (9.34, 30.94)	21.63 (9.21, 34.25)	14.91 (9.34, 26.71)	0.141
IL-6, pg./mL	75.98 (60.12, 104.92)	79.58 (60.77, 107.82)	71.66 (58.13, 102.79)	0.416
Lactate, mmol/L	4.84 (2.83, 8.94)	5.22 (2.66, 7.73)	4.38 (2.84, 10.60)	0.848
White blood cell, ×10^9^/L	13.77 (9.79, 19.70)	14.01 (10.24, 20.30)	13.18 (9.06, 19.59)	0.527
Hs-TnI, μg/L	1.66 (0.53, 8.81)	2.39 (0.66, 8.01)	1.21 (0.13, 13.58)	0.378
Poor-outcome, *n* (%)	99 (82.5%)	69 (82.1%)	30 (83.3%)	0.875

Data are presented as median (interquartile range) or *n* (percentile), unless specified otherwise. CPR, cardiopulmonary resuscitation; Hs-TnI, High sensitivity troponin I; IL-6, interleukin-6; NSE, neuron-specific enolase; SOFA, Sequential Organ Failure Assessment; sTREM-1, soluble triggering receptors expressed on myeloid cell-1; sTREM-2, soluble triggering receptors expressed on myeloid cell-2.

### 3.6 Model interpretation

**Figure 6:**
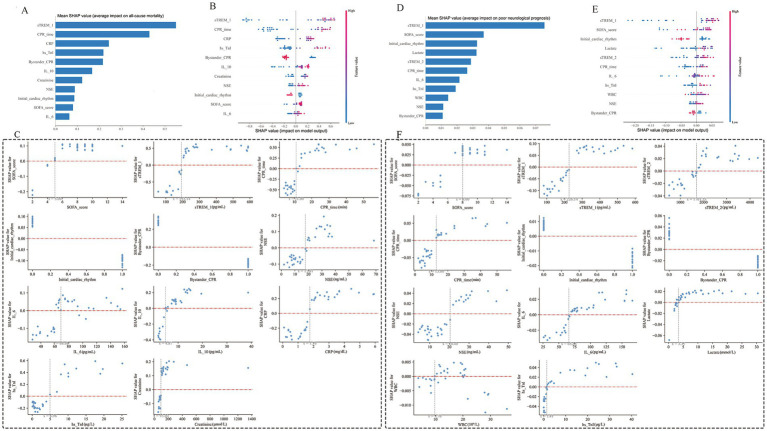
Global model explanation by the SHAP method of XGBoost **(A–C)** and RF **(D–F)** prediction models. **(A,D)** Importance score ranking of the model characteristics; **(B,E)** SHAP summary dot plot; **(C,F)** SHAP dependence plot. CPR, cardiopulmonary resuscitation; CRP, C-reactive protein; hs-TnI, High sensitivity troponin I; IL-6, Interleukin-6; IL-10, Interleukin-10; NSE, neuron-specific enolase; RF, Random Forest; SOFA, Sequential Organ Failure Assessment; SHAP, Shapley Additive explanation; sTREM-1, soluble triggering receptors expressed on myeloid cells 1; sTREM-2, soluble triggering receptors expressed on myeloid cells 2; WBC, White blood cell; XGBoost, eXtreme Gradient Boosting.

The SHAP method was employed to interpret XGBoost and RF models outputs by quantifying the contribution of individual features for each prediction. Figures 6B,E illustrated the importance of the SHAP features for the XGBoost and RF models, respectively. For instance, for CA patients with a high sTREM-1 level (in red), the 28-day all-cause mortality and 3-month neurological prognosis were likely unfavorable (right side). As shown in SHAP summary plots (Figures 6A,D), the contributions of the feature to the model were evaluated using the mean SHAP values and exhibited in descending order. The most related factor for 28-day all-cause mortality was sTREM-1. The top five pivotal features for 3-month neurological prognosis were sTREM-1, SOFA score, initial cardiac rhythm, lactate, and sTREM-2. The real values versus the SHAP values of these eleven features were shown in Figures 6C,F. The SHAP values higher than zero corresponded to a positive prediction in the model (a higher SHAP value indicated a higher risk of 28-day all-cause mortality and 3-month poor neurological prognosis). For instance, patient with a sTREM-1 ≥ 193.14 pg./mL had SHAP values higher than zero, which pushed the decision towards the “Non-survivors” class (Figure 6C). In addition, a high sTREM-1 level ≥ 178.83 pg./mL or a sTREM-2 level ≥1304.61 pg./mL pushed the decision towards the “Poor neurological outcome” class (Figure 6F).

**Figure 7:**
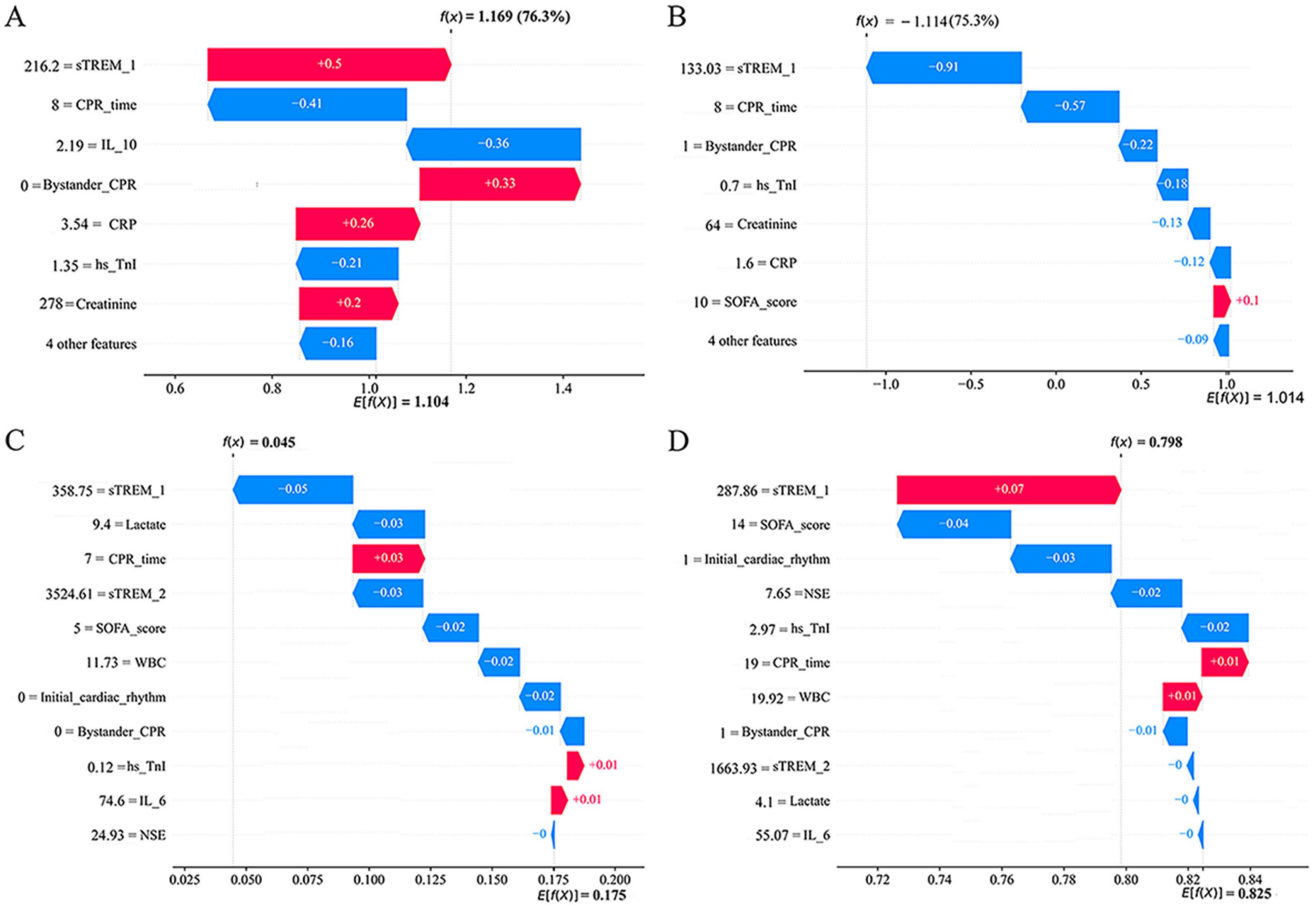
Local model explanation by the SHAP method of XGBoost **(A,B)** and RF **(C,D)** prediction models. Waterfall plot and evolution of risks contributed for individual patient at high **(A,C)** or low **(B,D)** risk of 28-day all-cause mortality **(A,B)** and 3-month poor neurological prognosis **(C,D)**. CPR, cardiopulmonary resuscitation; CRP, C-reactive protein; hs-TnI, High sensitivity troponin I; IL-6, Interleukin-6; IL-10, Interleukin-10; NSE, neuron-specific enolase; RF, Random Forest; SOFA, Sequential Organ Failure Assessment; SHAP, Shapley Additive explanation; sTREM-1, soluble triggering receptors expressed on myeloid cells 1; sTREM-2, soluble triggering receptors expressed on myeloid cells 2; WBC, White blood cell; XGBoost, eXtreme Gradient Boosting.

To enhance comprehension decision-making process of the two models at the individual level, we conducted a detailed interpretability analysis on four representative samples, as illustrated in Figure 7. Figure 7A showed a patient with sTREM-1 of 216.2 pg./mL, without bystander CPR, whose CPR time, IL-10, CRP, hs-TnI and creatinine concentrations were 8 min, 2.19 pg./mL, 3.54 mg/dL, 1.35 μg/L and 278 μmol/L, respectively. In this patient, the estimated likelihood of 28-day all-cause mortality was 76.3%.

## 4 Discussion

Our results demonstrated that both sTREM-1 and sTREM-2 levels were significantly increased after ROSC, with a lower level in the survivors compared with non-survivors. Meanwhile, sTREM-1 emerged as the top-ranked predictor in ML models for both 28-day mortality and 3-month poor neurological outcomes, outperforming traditional biomarkers. Moreover, the developed XGBoost and RF models demonstrated superior predictive accuracy compared to conventional clinical scoring systems, providing novel decision-support tools for post-resuscitation care. Additionally, we conducted interpretability analyses based the developed ML models.

The observed systemic inflammatory surge, with the elevated inflammatory mediators (TNF-*α*, IL-10, IL-6, and HMGB1) after ROSC, aligns with previous reports of ischemia–reperfusion mediated cytokine storm (29, 30). TREM-1 and TREM-2 are highly expressed in myeloid lineage cells and participate in various pathophysiological processes, such as sepsis and neurodegenerative disorders (7, 13, 31–33). Activation of TREM-1 triggers the gene expression to augment the inflammatory response, whereas TREM-2 can function to ameliorate the inflammatory responses (13, 31). Membrane-bound TREM-1 and TREM-2 are proteolytically cleaved by MMP and released into the extracellular space in the form of sTREM-1 and sTREM-2 (17, 34, 35). Therefore, sTREM-1 and sTREM-2 are considered to act as the regulator of the inflammatory status (17, 36). In our study, both sTREM-1 and sTREM-2 levels were elevated in CA patient with significant higher levels in the non-survivors compared with the survivors. In addition, we also found serum sTLR-4 and MMP-9 levels were significantly higher in non-survivors than those in survivors after ROSC. These results indicated a possible activation of TREM-1 and TREM-2 signaling pathways after ROSC, which could contribute to the systemic ischemia–reperfusion injury after ROSC (12, 36). This was evidenced by our observations that serum sTREM-1 and sTREM-2 were positively correlated with serum sTLR-4, MMP-9, NSE, IL-10, IL-6, and TNF-α within the first week after ROSC. These results were in line with previous research findings that the TREM-1/TREM-2/TLR-4 signaling pathways was activated and accompanied by the release of inflammatory mediators in patients after ROSC (10, 37).

Several studies have identified sTREM-1 and sTREM-2 as potential prognostic indicators in various inflammatory and non-inflammatory conditions (18, 20, 22, 36, 38, 39). In the current study, we observed that the non-survivors had significantly higher serum concentrations of sTREM-1 and sTREM-2 than the survivors. Meanwhile, high sTREM-1 and sTREM-2 levels were closely correlated with the disease severity indicators, including NSE, APACHE score, and SOFA score. Furthermore, SHAP values from our ML models suggest that sTREM-1 ranked first contribute to the occurrence of death and poor neurological outcome, while serum sTREM-2 only ranked fifth in ML model for poor neurological outcome. This difference can be partially explained by the predominant expression of TREM-2 in microglia and more sensitive predictive performance of cerebrospinal fluid sTREM-2 level on outcomes of non-inflammatory conditions (22, 40, 41). However, serum sTREM-1 is more commonly used as a potential independent predictor in both infectious or non-infectious diseases (20, 39), which may be associated with its up-regulative effect on inflammation. Along with prior evidence (42), sTREM-1 in our cohort demonstrated moderate prognostic value as a standalone biomarker, but its performance was significantly enhanced when incorporated into a multi-biomarker panel integrated with clinical variables in our ML model. In addition, traditional biomarkers like NSE and lactate showed reduced feature importance compared to sTREM-1 in SHAP analysis, suggesting TREM pathway activation may capture broader pathophysiological processes.

Early identification of CA patients with undesirable prognosis could tailor the targeted therapy. However, single-method diagnoses, including traditional clinical scores, radiological examinations and biomarkers, have limited accuracy. In our complex XGBoost model, we found that CA patients with high sTREM-1, CPR time, CRP, hs-TnI, IL-10, creatinine, NSE, IL-6 and SOFA score, and with non-shockable rhythm and without bystander CPR were positively associated with the 28-day all-cause mortality. Meanwhile, CA patients with high sTREM-1, lactate, sTREM-2, CPR time, IL-6, hs-TnI, WBC, NSE and SOFA score, and with non-shockable rhythm and without bystander CPR had poor 3-month neurological outcome in RF model. Only a few studies had employed ML models to predict prognosis of patients after ROSC, particularly in prospective cohort studies (43–45). The validation process revealed the high accuracy and stability of the XGBoost and RF models, which were further confirmed by internal validations.

## 5 Limitations

While our findings were strengthened by rigorous ML validation, several limitations merit consideration. First, this study was performed at a single institution with a modest number of patients, which could limit the generalizability of our findings. Second, we did not measure changes of sTREM-1 and sTREM-2 in the cerebrospinal fluid due to the difficulty in sample collection. Third, only internal validation was performed; more multicentered research with a large sample size are needed for external validation. Moreover, detailed variables related to post-cardiac arrest management—such as specific therapeutic interventions and physiological monitoring data—were not systematically collected. Future multicenter studies should integrate multimodal data, including electroencephalography, and brain imaging, to refine predictive algorithms. Finally, mechanistic studies exploring the exact pathophysiological role of TREM-1 and TREM-2 in CA-induced organ failure are warranted.

## 6 Conclusion

Serum sTREM-1 had better predictive value than sTREM-2 for 28-day all-cause mortality and 3-month poor neurological outcome. Furthermore, the newly developed XGBoost and RF models incorporating sTREM-1 and/or sTREM-2 demonstrated superior predictive accuracy compared to conventional clinical scoring systems. These findings positioned sTREM-1 and sTREM-2 as novel biomarkers for post-resuscitation outcomes while establishing ML prediction models as a superior decision-support framework in critical care settings.

## Data Availability

The original contributions presented in the study are included in the article/Supplementary material, further inquiries can be directed to the corresponding authors.

## Supplementary material

SUPPLEMENTARY FIGURE S1 The performances of XGBoost **(A–D)** and RF **(E–H)** prediction models. **(A,E)** Precision-recall curve; **(B,F)** Calibration curve; **(C,G)** decision curve analysis; **(D,H)** Confusion matrix. AP, area under the precision-recall curve; DCA, decision curve analysis; PR, precision-recall; RF, Random Forest; XGBoost, eXtreme Gradient Boosting.
