# Supplementary figures and images for: Associations of serum sTREM-1 and sTREM-2 with mortality and neurological prognosis in patients resuscitated from cardiac arrest: a machine learning-based approach
Source: Front Med (Lausanne). 2026 Mar 3;13:1717571. doi: 10.3389/fmed.2026.1717571 (PMC12992311; doi:10.3389/fmed.2026.1717571)

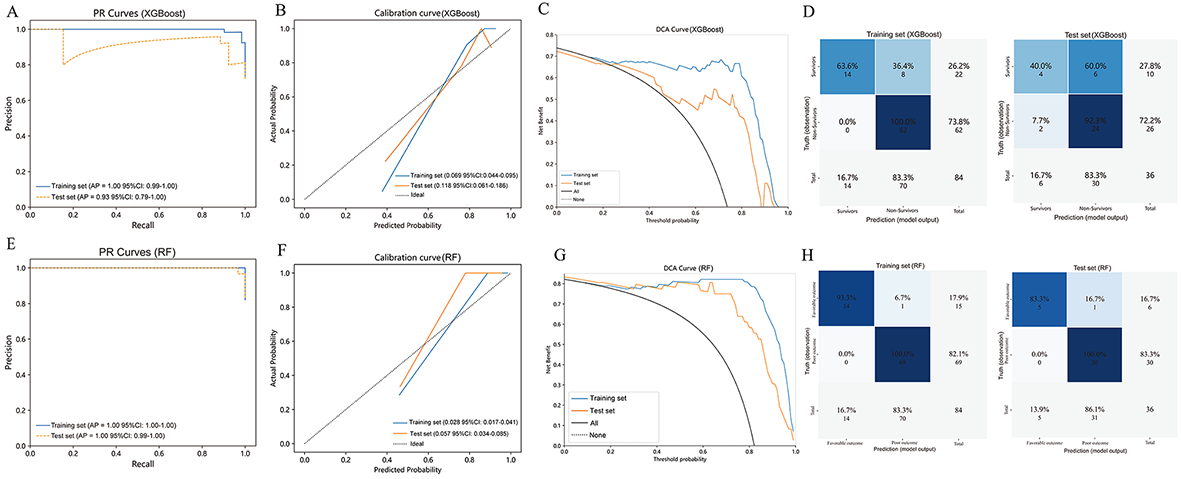

Supplement: SUPPLEMENTARY FIGURE S1 — The performances of XGBoost (A–D) and RF (E–H) prediction models. (A,E) Precision-recall curve; (B,F) Calibration curve; (C,G) decision curve analysis; (D,H) Confusion matrix. AP, area under the precision-recall curve; DCA, decision curve analysis; PR, precision-recall; RF, Random Forest; XGBoost, eXtreme Gradient Boosting. [file Image_1.tif]
