# Supplementary material for: Associations of serum sTREM-1 and sTREM-2 with mortality and neurological prognosis in patients resuscitated from cardiac arrest: a machine learning-based approach
Source: Front Med (Lausanne). 2026 Mar 3;13:1717571. doi: 10.3389/fmed.2026.1717571 (PMC12992311; doi:10.3389/fmed.2026.1717571)
Supplement: Supplementary file 2 [file Table_1.docx]

**Table S1** Optimal Hyperparameter Configurations for Machine Learning Models.

| **Model** | **Description** |
| --- | --- |
| **28-day all-cause mortality** |  |
| Logistic Regression | 'C': 0.003927106263955029, 'penalty': 'l2', 'solver': 'saga', 'class_weight': 'balanced', 'max_iter': 499 |
| Random Forest | 'criterion': 'gini', 'max_depth': None, 'min_samples_leaf': 5, 'min_samples_split': 2, 'n_estimators': 100 |
| Gaussian Naive Bayes | Default |
| Support Vector Machine | 'C': 10 |
| K-Nearest Neighbor | 'algorithm': 'auto', 'metric': 'manhattan', 'n_neighbors': 9, 'weights': 'uniform' |
| eXtreme Gradient Boosting | 'learning_rate': 0.01, 'max_depth': 4, 'n_estimators': 400 |
| Light Gradient Boosting Machine | 'learning_rate': 0.05, 'max_bin': 255, 'n_estimators': 25, 'num_leaves': 7, 'num_threads': 1 |
| Decision Tree | 'ccp_alpha': 0.05, 'criterion': 'entropy', 'max_depth': 5, 'min_samples_leaf': 2, 'min_samples_split': 5, 'splitter': 'random' |
| **3-month neurological prognosis** |  |
| Logistic Regression | 'C': 0.0003314459707751232, 'penalty': 'l2', 'solver': 'saga', 'class_weight': 'balanced', 'max_iter': 276 |
| Random Forest | 'criterion': 'entropy', 'max_depth': None, 'min_samples_leaf': 1, 'min_samples_split': 5, 'n_estimators': 200 |
| Gaussian Naive Bayes | Default |
| Support Vector Machine | 'C': 100 |
| K-Nearest Neighbor | 'algorithm': 'auto', 'metric': 'manhattan', 'n_neighbors': 9, 'weights': 'distance' |
| eXtreme Gradient Boosting | 'learning_rate': 0.02, 'max_depth': 3, 'n_estimators': 500 |
| Light Gradient Boosting Machine | 'learning_rate': 0.05, 'max_bin': 255, 'n_estimators': 50, 'num_leaves': 7, 'num_threads': 1 |
| Decision Tree | 'ccp_alpha': 0.05, 'criterion': 'gini', 'max_depth': 3, 'min_samples_leaf': 4, 'min_samples_split': 2, 'splitter': 'best' |
