# Supplementary material for: Associations of serum sTREM-1 and sTREM-2 with mortality and neurological prognosis in patients resuscitated from cardiac arrest: a machine learning-based approach
Source: Front Med (Lausanne). 2026 Mar 3;13:1717571. doi: 10.3389/fmed.2026.1717571 (PMC12992311; doi:10.3389/fmed.2026.1717571)
Supplement: Supplementary file 3 [file Table_2.docx]

**Table S****2** Comparisons of baseline characteristics between patients with favorable and poor neurological outcome

| **Characteristics** | **Healthy volunteers** | **Favorable outcome** | **Poor outcome** | ***P*** |
| --- | --- | --- | --- | --- |
|  | **(*n* = 30)** | **(*n* = 21)** | **(*n* = 99)** |  |
| Age, years | 62.5 (49.5, 75.5) | 73.0 (54.5, 81.0) | 69.0 (58.0, 77.0) | 0.334 |
| Male, *n* (%) | 19 (63.3%) | 16 (76.2%) | 62 (62.6%) | 0.491 |
| Past medical history, *n* (%) |  |  |  |  |
| Diabetes | — | 7 (33.3%) | 22 (22.2%) | 0.276 |
| Coronary heart disease | — | 3 (14.3%) | 18 (18.2%) | 0.670 |
| Hypertension | — | 14 (66.7%) | 51 (51.5%) | 0.236 |
| Chronic kidney disease | — | 3 (14.3%) | 6 (6.1%) | 0.192 |
| Chronic pulmonary disease | — | 4 (19.0%) | 14 (14.1%) | 0.518 |
| Post-operation | — | 3 (14.3%) | 18 (18.2%) | 0.670 |
| Cardiac arrest cause, *n* (%) |  |  |  | 0.643 |
| Respiratory | — | 7 (33.3%) | 24 (24.2%) | — |
| Cardiac | — | 7 (33.3%) | 37 (37.4%) | — |
| Cerebral | — | 4 (19.0%) | 14 (14.1%) | — |
| Others | — | 3 (14.3%) | 24 (24.2%) | — |
| Cardiac arrest location, *n* (%) |  |  |  | 0.445 |
| Out of hospital | — | 5 (23.8%) | 35 (35.4%) | — |
| In hospital | — | 16 (76.2%) | 64 (64.6%) | — |
| Bystander CPR, *n* (%) | — | 17 (80.9%) | 55 (55.6%)^a^ | 0.031 |
| Initial cardiac rhythm, *n* (%) |  |  |  | 0.004 |
| Shockable rhythm | — | 14 (66.7%) | 33 (33.3%)^a^ | — |
| Non-shockable rhythm | — | 7 (33.3%) | 66 (66.7%)^a^ | — |
| CPR time, min | — | 8.0 (5.0, 11.5) | 15.0 (8.0, 28.0)^a^ | 0.005 |
| Length of ICU stay, days | — | 13.0 (10.5, 16.5) | 4.0 (1.0, 9.0)^a^ | <0.001 |
| Treatments, *n* (%) |  |  |  |  |
| Renal replacement therapy | — | 7 (33.3%) | 32 (32.3%) | 0.928 |
| Mechanical ventilation | — | 19 (90.5%) | 99 (100%) | 0.029 |
| Hemodynamic support | — | 19 (90.5%) | 99 (100%) | 0.029 |
| Laboratory findings |  |  |  |  |
| White blood cell, ×10^9^/L | 6.83 (5.46, 7.92) | 12.26 (8.28, 21.43)^b^ | 14.05 (10.40, 19.61)^b^ | <0.001 |
| Neutrophils, ×10^9^/L | 4.36 (3.28, 5.12) | 7.87 (5.86, 17.22)^b^ | 10.29 (6.90, 15.63)^b^ | <0.001 |
| C-reactive protein, mg/dL | 0.23 (0.09, 0.32) | 1.55 (0.95, 2.33)^b^ | 2.03 (1.14, 3.42)^b^ | <0.001 |
| Procalcitonin, ng/mL | 0.21 (0.08, 0.31) | 0.56 (0.05, 10.14)^b^ | 0.77 (0.13, 6.23)^b^ | 0.001 |
| Creatinine, μmol/L | 72.00 (60.75, 80.25) | 87.00 (64.50, 119.00)^b^ | 102.00 (80.00, 162.00)^b^ | <0.001 |
| AST, IU/L | 32.00 (20.50, 39.25) | 47.00 (27.00, 132.00)^b^ | 107.00 (47.00, 233.00)^ab^ | <0.001 |
| ALT, IU/L | 24.50 (17.75, 34.25) | 49.00 (16.00, 120.00)^b^ | 67.00 (35.00, 147.00)^ab^ | <0.001 |
| Lactate, mmol/L | 0.23 (0.14, 0.52) | 2.58 (1.22, 4.04)^b^ | 5.70 (3.29, 11.00)^ab^ | <0.001 |
| Hs-TnI, μg/L | 0.001 (0.000, 0.010) | 1.560 (0.595, 5.470)^b^ | 1.940 (0.450, 9.740)^b^ | <0.001 |
| BNP, pg/mL | 28.54 (20.68, 44.61) | 124.00 (63.29, 540.49)^b^ | 296.20 (85.32, 1004.70)^b^ | <0.001 |
| APACHE II score | — | 16.0 (8.5, 24.0) | 22.0 (17.0, 26.0)^a^ | 0.027 |
| SOFA score | — | 3.0 (2.0, 6.5) | 8.0 (5.0, 9.0)^a^ | <0.001 |

Data are presented as median (interquartile range) or *n* (percentile), unless specified otherwise. ^a^*P* < 0.05 compared with favorable outcome; ^b^*P* < 0.05 compared with healthy volunteers.

APACHE II Acute Physiology and Chronic Health Evaluation II, ALT alanine aminotransferase, AST aspartate aminotransferase, BNP brain natriuretic peptide, CPR cardiopulmonary resuscitation, Hs-TnI High sensitivity troponin I, ICU intensive care unit, SOFA Sequential Organ Failure Assessment.
