# Supplementary material for: Associations of serum sTREM-1 and sTREM-2 with mortality and neurological prognosis in patients resuscitated from cardiac arrest: a machine learning-based approach
Source: Front Med (Lausanne). 2026 Mar 3;13:1717571. doi: 10.3389/fmed.2026.1717571 (PMC12992311; doi:10.3389/fmed.2026.1717571)
Supplement: Supplementary file 4 [file Table_3.docx]

**Table S3** Comparisons of biomarkers between patients with favorable and poor neurological outcome

| **Biomarkers** | **Healthy volunteers** | **Favorable outcome** | **Poor outcome** | ***P*** |
| --- | --- | --- | --- | --- |
|  | **(*n* = 30)** | **(*n* = 21)** | **(*n* = 99)** |  |
| **Day 1 after ROSC** |  |  |  |  |
| sTREM-1, pg/mL | 15.79 (8.32, 22.24) | 133.03 (94.29, 187.18)^b^ | 270.59 (193.14, 438.39)^ab^ | < 0.001 |
| sTREM-2, pg/mL | 304.18 (141.27, 413.72) | 1016.82 (681.29, 1291.37)^b^ | 1880.07 (1048.81, 2916.11)^ab^ | < 0.001 |
| sTLR-4, ng/mL | 0.21 (0.09, 1.03) | 2.13 (1.74, 3.84)^b^ | 4.79 (3.22, 7.69)^ab^ | < 0.001 |
| MMP-9, ng/mL | 18.05 (10.06, 29.86) | 90.27 (44.78, 154.88)^b^ | 137.82 (76.57, 258.50)^ab^ | < 0.001 |
| NSE, ng/mL | 4.56 (2.18, 7.50) | 12.92 (8.74, 20.49)^b^ | 21.26 (9.74, 32.85)^ab^ | < 0.001 |
| IL-6, pg/mL | 22.59 (14.82, 28.25) | 62.17 (43.86, 91.39)^b^ | 78.37 (62.00, 110.75)^b^ | < 0.001 |
| IL-10, pg/mL | 1.84 (0.77, 3.04) | 7.34 (2.97, 11.20)^b^ | 8.80 (4.75, 13.83)^b^ | < 0.001 |
| TNF-α, pg/mL | 6.04 (2.75, 9.59) | 48.45 (23.15, 73.86)^b^ | 66.81 (43.44, 109.69)^b^ | < 0.001 |
| HMGB1, ng/mL | 6.72 (4.66, 9.62) | 7.22 (4.82, 13.64) | 8.69 (6.03, 12.00) | 0.119 |
| **Day 3 after ROSC** | **(*n* = 30)** | **(*n* = 21)** | **(*n* = 61)** |  |
| sTREM-1, pg/mL | 15.79 (8.32, 22.24) | 151.36 (95.94, 183.47)^b^ | 152.63 (116.39, 200.28)^b^ | < 0.001 |
| sTREM-2, pg/mL | 304.18 (141.27, 413.72) | 1519.72 (946.58, 2128.12)^b^ | 1506.33 (958.88, 1924.62)^b^ | < 0.001 |
| sTLR-4, ng/mL | 0.21 (0.09, 1.03) | 2.60 (1.67, 4.36)^b^ | 3.74 (2.39, 5.97)^b^ | < 0.001 |
| MMP-9, ng/mL | 18.05 (10.06, 29.86) | 70.37 (40.01, 126.36)^b^ | 130.91 (76.30, 217.37)^ab^ | < 0.001 |
| NSE, ng/mL | 4.56 (2.18, 7.50) | 9.98 (8.01, 18.94)^b^ | 25.01 (8.07, 41.63)^ab^ | < 0.001 |
| IL-6, pg/mL | 22.59 (14.82, 28.25) | 72.33 (48.27, 96.82)^b^ | 84.32 (60.99, 108.69)^b^ | < 0.001 |
| IL-10, pg/mL | 1.84 (0.77, 3.04) | 4.84 (3.52, 10.09)^b^ | 7.71 (5.02, 13.07)^b^ | < 0.001 |
| TNF-α, pg/mL | 6.04 (2.75, 9.59) | 50.25 (24.68, 72.00)^b^ | 61.44 (37.45, 98.51)^b^ | < 0.001 |
| HMGB1, ng/mL | 6.72 (4.66, 9.62) | 32.56 (15.13, 45.01)^b^ | 43.86 (16.99, 62.56)^b^ | < 0.001 |
| **Day 5 after ROSC** | **(*n* = 30)** | **(*n* = 21)** | **(*n* = 38)** |  |
| sTREM-1, pg/mL | 15.79 (8.32, 22.24) | 117.58 (93.16, 180.75)^b^ | 166.76 (84.99, 217.43)^b^ | < 0.001 |
| sTREM-2, pg/mL | 304.18 (141.27, 413.72) | 1052.59 (856.23, 1456.56)^b^ | 1077.67 (800.56, 1882.94)^b^ | < 0.001 |
| sTLR-4, ng/mL | 0.21 (0.09, 1.03) | 2.35 (1.56, 4.24)^b^ | 2.15 (1.68, 3.64)^b^ | < 0.001 |
| MMP-9, ng/mL | 18.05 (10.06, 29.86) | 90.07 (55.98, 166.41)^b^ | 142.98 (63.91, 225.68)^b^ | < 0.001 |
| NSE, ng/mL | 4.56 (2.18, 7.50) | 8.39 (3.01, 26.68)^b^ | 20.76 (8.59, 45.48)^b^ | < 0.001 |
| IL-6, pg/mL | 22.59 (14.82, 28.25) | 62.75 (38.49, 90.98)^b^ | 65.70 (35.86, 95.03)^b^ | < 0.001 |
| IL-10, pg/mL | 1.84 (0.77, 3.04) | 5.05 (2.45, 9.20)^b^ | 7.6300 (3.90, 13.86)^b^ | < 0.001 |
| TNF-α, pg/mL | 6.04 (2.75, 9.59) | 2.35 (1.56, 4.24)^b^ | 2.15 (1.68, 3.64)^b^ | < 0.001 |
| HMGB1, ng/mL | 6.72 (4.66, 9.62) | 17.63 (8.67, 23.85)^b^ | 28.16 (12.28, 38.89)^b^ | < 0.001 |

Data are presented as median (interquartile range) or *n* (percentile), unless specified otherwise. ^a^*P* < 0.05 compared with favorable outcomes; ^b^*P* < 0.05 compared with healthy volunteers.

HMGB1 high mobility group protein 1, IL-6 Interleukin‐6, IL-10 Interleukin‐10, MMP-9 matrix metalloproteinase-9, NSE neuron-specific enolase, ROSC restoration of spontaneous circulation, sTREM-1 soluble triggering receptors expressed on myeloid cells-1, sTREM-2 soluble triggering receptors expressed on myeloid cells-2, sTLR-4 soluble toll-like receptor 4, TNF‐α tumor necrosis factor‐α.
