# Supplementary material for: Associations of serum sTREM-1 and sTREM-2 with mortality and neurological prognosis in patients resuscitated from cardiac arrest: a machine learning-based approach
Source: Front Med (Lausanne). 2026 Mar 3;13:1717571. doi: 10.3389/fmed.2026.1717571 (PMC12992311; doi:10.3389/fmed.2026.1717571)
Supplement: Supplementary file 5 [file Table_4.doc]

**Table S4** Potential variables included in LASSO regression analysis

| **Number** | **Variables** |
| --- | --- |
| 1 | Sex |
| 2 | Age |
| 3 | CPR time |
| 4 | Initial cardiac rhythm |
| 5 | Cardiac arrest cause |
| 6 | Cardiac arrest location |
| 7 | Bystander CPR |
| 8 | APACHE II score |
| 9 | SOFA score |
| 10 | sTREM-1 |
| 11 | sTREM-2 |
| 12 | NSE |
| 13 | TNF-α |
| 14 | IL-6 |
| 15 | IL-10 |
| 16 | HMGB1 |
| 17 | Lactate |
| 18 | CRP |
| 19 | WBC |
| 20 | Neutrophils |
| 21 | AST |
| 22 | ALT |
| 23 | hs-TnI |
| 24 | BNP |
| 25 | PCT |
| 26 | Creatinine |

APACHE II Acute Physiology and Chronic Health Evaluation II, ALT alanine aminotransferase, AST aspartate aminotransferase, BNP brain natriuretic peptide, CPR cardiopulmonary resuscitation, CRP C-reactive protein, HMGB1 high mobility group protein 1, hs-TnI High sensitivity troponin I, IL-6 Interleukin‐6, IL-10 Interleukin‐10, LASSO least absolute shrinkage and selection operator, NSE neuron-specific enolase, PCT Procalcitonin, ROSC restoration of spontaneous circulation, SOFA Sequential Organ Failure Assessment, sTREM-1 soluble triggering receptors expressed on myeloid cells 1, sTREM-2 soluble triggering receptors expressed on myeloid cells 2, sTLR-4 soluble toll-like receptor 4, TNF‐α tumor necrosis factor‐α, WBC White blood cell.
