# Supplementary material for: Associations of serum sTREM-1 and sTREM-2 with mortality and neurological prognosis in patients resuscitated from cardiac arrest: a machine learning-based approach
Source: Front Med (Lausanne). 2026 Mar 3;13:1717571. doi: 10.3389/fmed.2026.1717571 (PMC12992311; doi:10.3389/fmed.2026.1717571)
Supplement: Supplementary file 6 [file Table_5.docx]

**Table S5** Comparative analysis of the performance outcomes across various machine learning model to predict 28-day all-cause mortality

| **Model** | **Accuracy (%)** | **Specificity (%)** | **Sensitivity (%)** | **NPV (%)** | **PPV (%)** | **F1 score (%)** | **Kappa score (%)** |
| --- | --- | --- | --- | --- | --- | --- | --- |
| **Training set** |  |  |  |  |  |  |  |
| LR | 75.0 | 54.5 | 82.3 | 82.3 | 83.6 | 82.9 | 36.3 |
| SVM | 84.5 | 54.5 | 95.2 | 95.2 | 85.5 | 90.1 | 55.4 |
| KNN | 80.9 | 54.5 | 90.3 | 90.3 | 84.8 | 87.5 | 47.7 |
| DT | 86.9 | 50.0 | 100.0 | 100.0 | 84.9 | 91.8 | 59.6 |
| RF | 97.6 | 90.9 | 100.0 | 100.0 | 96.9 | 98.4 | 93.7 |
| LightGBM | 85.7 | 45.5 | 100.0 | 100.0 | 83.8 | 91.2 | 55.2 |
| GNB | 76.2 | 81.8 | 74.2 | 74.2 | 92.0 | 82.1 | 47.6 |
| XGBoost | 90.5 | 63.6 | 100.0 | 100.0 | 88.6 | 93.9 | 72.1 |
| **Test set** |  |  |  |  |  |  |  |
| LR | 72.2 | 40.0 | 84.6 | 84.6 | 78.5 | 81.5 | 26.2 |
| SVM | 83.3 | 60.0 | 92.3 | 92.3 | 85.7 | 88.9 | 55.7 |
| KNN | 77.8 | 70.0 | 80.8 | 80.7 | 87.5 | 84.0 | 47.8 |
| DT | 80.6 | 50.0 | 92.3 | 92.3 | 82.8 | 87.3 | 46.6 |
| RF | 77.8 | 40.0 | 92.3 | 92.3 | 80.0 | 85.8 | 36.8 |
| LightGBM | 80.6 | 40.0 | 96.2 | 96.2 | 80.6 | 87.7 | 42.7 |
| GNB | 83.3 | 80.0 | 84.6 | 84.6 | 91.7 | 88.0 | 60.9 |
| XGBoost | 77.8 | 40.0 | 92.3 | 92.3 | 80.0 | 85.7 | 36.8 |

DT Decision Tree, GNB Gaussian Naive Bayes, KNN K-Nearest Neighbor, LightGBM Light Gradient Boosting Machine, LR Logistic Regression, NPV negative predictive value, PPV positive predictive value, RF Random Forest, SVM Support Vector Machine, XGBoost eXtreme Gradient Boosting.
