# Supplementary material for: Associations of serum sTREM-1 and sTREM-2 with mortality and neurological prognosis in patients resuscitated from cardiac arrest: a machine learning-based approach
Source: Front Med (Lausanne). 2026 Mar 3;13:1717571. doi: 10.3389/fmed.2026.1717571 (PMC12992311; doi:10.3389/fmed.2026.1717571)
Supplement: Supplementary file 7 [file Table_6.docx]

**Table S6** Comparative analysis of the performance outcomes across various machine learning model to predict 3-month neurological prognosis

| **Model** | **Accuracy (%)** | **Specificity (%)** | **Sensitivity (%)** | **NPV (%)** | **PPV (%)** | **F1 score (%)** | **Kappa score (%)** |
| --- | --- | --- | --- | --- | --- | --- | --- |
| **Training set** |  |  |  |  |  |  |  |
| LR | 77.4 | 46.7 | 84.1 | 84.1 | 87.9 | 85.9 | 28.5 |
| SVM | 82.1 | 0.00 | 100.0 | 100.0 | 82.1 | 90.2 | 0.00 |
| KNN | 88.1 | 33.3 | 100.0 | 100.0 | 87.3 | 93.2 | 45.1 |
| DT | 88.1 | 60.0 | 94.2 | 94.2 | 91.5 | 92.8 | 57.2 |
| RF | 98.8 | 93.3 | 100.0 | 100.0 | 98.6 | 99.3 | 95.8 |
| LightGBM | 100.0 | 100.0 | 100.0 | 100.0 | 100.0 | 100.0 | 100.0 |
| GNB | 92.8 | 86.7 | 94.2 | 94.2 | 97.0 | 95.6 | 76.9 |
| XGBoost | 96.4 | 80.0 | 100.0 | 100.0 | 95.8 | 97.9 | 86.8 |
| **Test set** |  |  |  |  |  |  |  |
| LR | 72.2 | 0 | 86.7 | 86.7 | 81.3 | 83.9 | -15.4 |
| SVM | 83.3 | 0 | 100.0 | 100.0 | 83.3 | 90.9 | 0.00 |
| KNN | 83.3 | 16.7 | 96.7 | 96.7 | 85.3 | 90.6 | 18.2 |
| DT | 88.9 | 50.0 | 96.7 | 96.7 | 90.6 | 93.5 | 53.8 |
| RF | 97.2 | 83.3 | 100.0 | 100.0 | 96.8 | 98.4 | 89.3 |
| LightGBM | 91.7 | 50.0 | 100.0 | 100.0 | 90.9 | 95.2 | 62.5 |
| GNB | 88.9 | 83.3 | 90.0 | 90.0 | 96.4 | 93.1 | 64.7 |
| XGBoost | 97.2 | 83.3 | 100.0 | 100.0 | 96.8 | 98.4 | 89.3 |

DT Decision Tree, GNB Gaussian Naive Bayes, KNN K-Nearest Neighbor, LightGBM Light Gradient Boosting Machine, LR Logistic Regression, NPV negative predictive value, PPV positive predictive value, RF Random Forest, SVM Support Vector Machine, XGBoost eXtreme Gradient Boosting.
